# Activity-Dependent Plasticity of Spinal Circuits in the Developing and Mature Spinal Cord

**DOI:** 10.1155/2012/964843

**Published:** 2012-08-01

**Authors:** Behdad Tahayori, David M. Koceja

**Affiliations:** Department of Kinesiology and Program in Neuroscience, Indiana University Bloomington, Bloomington, IN 47405-7109, USA

## Abstract

Part of the development and maturation of the central nervous system (CNS) occurs through interactions with the environment. Through physical activities and interactions with the world, an animal receives considerable sensory information from various sources. These sources can be internally (proprioceptive) or externally (such as touch and pressure) generated senses. Ample evidence exists to demonstrate that the sensory information originating from large diameter afferents (Ia fibers) have an important role in inducing essential functional and morphological changes for the maturation of both the brain and the spinal cord. The Ia fibers transmit sensory information generated by muscle activity and movement. Such use or activity-dependent plastic changes occur throughout life and are one reason for the ability to acquire new skills and learn new movements. However, the extent and particularly the mechanisms of activity-dependent changes are markedly different between a developing nervous system and a mature nervous system. Understanding these mechanisms is an important step to develop strategies for regaining motor function after different injuries to the CNS. Plastic changes induced by activity occur both in the brain and spinal cord. This paper reviews the activity-dependent changes in the spinal cord neural circuits during both the developmental stages of the CNS and in adulthood.

## 1. Introduction

Deprivation of sensory information during certain periods of an animal's life span causes substantial impairment in the normal development and function of the central nervous system (CNS). Hence, this period is referred to as a critical period. The pioneering works of Hubel and Wiesel [1–5], which culminated in the Nobel Prize in Medicine, showed that during a critical period, depriving kittens' visual information for as few as three to four days resulted in a substantial decline in the number of striatal neurons [1]. Other studies have shown that during normal development the acquisition of motor abilities such as standing [6] and walking [7] are extensively dependent on various sensory inputs generated by movement. The term *activity-dependent plasticity* is used to describe the changes induced in the CNS associated with movement activity. These activity-dependent changes occur ubiquitously in the CNS; connections between the brain and spinal neurons and connections between sensory neurons and motoneurons of the spinal cord also show extensive reorganization in response to movement and activity.

However, activity-dependent changes in the nervous system are not solely limited to the developing period but also exist throughout the life span for both the spinal cord [8] and the brain [9]. The adult CNS also undergoes plastic changes during the learning of new motor skills which persists for extended periods of time. Conversely, the loss of plasticity of the nervous system with aging has been shown to be related to the decline in specific motor capacities of the individual. For example, a decline in the flexibility or adaptability of spinal reflexes has been shown in different and independent studies to be meaningfully correlated with fall risk or abnormal postural control strategies [10–14]. In this instance, regaining the adaptive capacity of the nervous system is, therefore, a promising strategy for neurological rehabilitation.

The purpose of this paper is to review and compare activity-dependent plasticity of spinal circuits during development and in adulthood, focusing on the fundamental differences in the mechanisms of spinal plasticity between development and adulthood. Understanding the underlying mechanisms involved in the activity-dependent induction of plasticity is potentially meaningful for modern treatments of a variety of movement disorders.

## 2. Activity-Dependent Plasticity during Development

A variety of mechanisms ranging from intrinsic cellular and morphological properties to genetic and epigenetic factors [17, 18] have been identified which participate in the transition of an immature nervous system into its final shape and function. The maturation process partly depends on the activity of the neonate during the movement development critical period [19]. For example, the ability of kittens to acquire standing, walking, and running skills has been shown to be related to the maturation of motor units and the central connectivity between the motoneurons and their various sensory inputs [20]. Studies of the past two decades have shown that during maturation, extensive morphological, molecular, and structural changes occur in motoneurons [21, 22]. In this part, we will review the effect of sensory input, receptor activity, and descending drive on the plastic changes of the spinal circuits during development.

### 2.1. The Importance of Sensory Input for Proper Development

Experimental studies have found molecular correlates with activity, which are predominantly observed during maturation and the developmental progression of motoneurons. One of the well-studied molecules is a monoclonal antibody which recognizes a certain proteoglycan known as Cat-301 proteoglycan. Expression of Cat-301 substantially increases in association with movement [26, 27]. This proteoglycan does not exist in immediate postnatal cells but its expression increases as development proceeds, and it has a substantial role in the morphological and physiological maturation of the motoneurons. There is evidence to show that this movement-associated increase in Cat-301 expression is actually related to the sensory input from large diameter fibers and not the generation of the movement per se. Studies with animal models have shown that crushing the sciatic nerve in neonatal hamsters seriously affects the expression of Cat-301 proteoglycan on the cell body of motoneurons [27] whereas in adulthood, a sciatic nerve crush injury does not cause a substantial change in the expression of Cat-301, emphasizing its importance during development. Severing the sensory afferents via dorsal rhizotomy during development also produces the same pattern of results in the expression of Cat-301, suggesting the importance of sensory input to the motoneuron. Interestingly, destruction of the smaller-diameter unmyelinated afferents (C-fibers) which are involved in the transmission of pain information, does not affect the expression of the antibody [26]. Taken together, it seems that the proprioceptive sensory information from muscle spindles, whose firing rates are related to the movement of body parts, has a significant role for the induction of change in the spinal circuits of developing animals.

### 2.2. NMDA Receptors Have an Important Role in the Induction of Plasticity

It is well known that sensory Ia fibers make both monosynaptic and oligosynaptic connections to alpha motoneurons in the ventral horn and comprise the reflex arc, using glutamine as the excitatory neurotransmitter. In the spinal cord, glutamine activates two major types of ionotropic receptors: NMDA and AMPA receptors. It was initially thought that these two types of glutamatergic ionotropic receptors have differential roles in synaptic transmission of the reflexes. Initial studies suggested that NMDA receptors were involved in the transmission of polysynaptic reflexes and AMPA receptors were involved in the transmission of the monosynaptic reflexes; however subsequent studies have shown this not to be the case [28]. Rather, numerous studies have shown that the NMDA receptor function is crucial for the induction of plasticity [29]. For example, in an *in vitro *experimental setup, Fields and colleagues cultured the spinal motoneurons of 13-d mouse fetuses in a 3-chambered cell-culture system and electrically stimulated the sensory afferents of the motoneurons of one of the chambers. It was shown that the motoneurons which were subject to chronic stimulation developed stronger synaptic connections with the afferent fibers in terms of yielding larger excitatory postsynaptic potentials (EPSP) compared to the untreated chambers of the sensory afferents. Therefore, this experimental model suggested that an increase in sensory input to spinal motoneurons could increase the efficacy of this synaptic connection. These stimulation-induced changes in sensory afferent efficacy were suppressed by the application of the selective NMDAR antagonist APV [30], suggesting that sensory input to motoneurons induced morphological changes through the activation of the NMDA receptors. Cell bodies and dendrites initially grow in size and number but after this initial growth, they show a regression until they reach their final mature configuration. NMDA receptors have a role in this dendritic growth and retraction. The application of an NMDA antagonist during the first three weeks after birth substantially abolishes motoneuron and dendritic growth in neonate hamsters; whereas in adulthood, NMDAR blockade has not been shown to affect motoneuron morphology [31]. In conclusion, although NMDA glutamatergic receptors do not have a significant role in signal transmission of the reflexes, NMDARs do have an important role in inducing plastic changes in spinal motoneurons.

Interestingly, NMDA receptors can be found throughout the spinal cord gray matter (ventral and dorsal horn) at very early stages of development [32], but during maturation they are essentially eliminated from all parts of the spinal cord except from the substantia gelatinosa [33]. NMDA receptors of the substantia gelatinosa have roles in modifying the input from sensory fibers such as A-delta. Experimental studies on rat spinal cord has shown that low-frequency stimulation of A-delta fibers can induce NMDA-dependent long-term depression (LTD) in substantia gelatinosa [34]. In pathologic conditions such as excitotoxicity, activation of these receptors in the CNS is a contributing part of the process of neuronal destruction [35]. Likewise, NMDA receptors might be involved in the development of neuropathic pain [36]. Although these receptors are eliminated from the ventral horn in the mature spinal cord, this does not rule out the ability for change at later stages of life.

### 2.3. Descending Inputs Are Essential for the Induction of Plasticity

The induction of temporary or permanent plastic change is, logically, also contingent on descending drive. For example, severing the spinal cord during the developmental stages substantially reduces the expression of Cat-301 on the motoneuron soma in neonatal hamsters [27]. At birth, the corticospinal tract makes synapses with both the dorsal and ventral regions of the spinal cord. However, during the course of development, the connections between the corticospinal tract and the ventral neurons are pruned. The pattern of synapse elimination seems to be complex and dependent on activation in both the contralateral and ipsilateral tracts. In human [37, 38], as well as monkeys [39] and mice [40], the corticospinal tract makes synaptic connections with both contralateral as well as ipsilateral spinal motoneurons, and during normal development of the CNS, the majority of the connections to the ipsilateral side are eliminated. Ablation of the cortex in subprimate mammals during the early stages of postnatal life has been shown to prevent the elimination of the corticospinal tract connections to the ipsilateral motoneurons [37]. This injury-induced maintenance of ipsilateral projections from the corticospinal tract is accompanied by a hypertrophy of the cortex of the undamaged side [40]. In line with animal studies, the same findings have been indirectly shown in human subjects. In newborns, the application of transcranial magnetic stimulation (TMS) to the cortex elicits bilateral muscle-twitch responses to both limbs with almost the same amplitude but with a shorter delay on the ipsilateral side. Studies on patients with cerebral palsy (nonprogressive damage to developing fetal or infant brain [41]) have also shown the same pathologies as those observed in animal models. In these patients, the bilateral pattern of innervation of the spinal motoneurons from the cortex persists and is not eliminated during maturation [42]. From a behavioral perspective, this lack of remodeling and selective elimination of the corticospinal tract connections could partly explain why children with cerebral palsy cannot tonically decrease the amplitude of the H-reflex (explained in Section 3, see below) during walking [43, 44]. In children with diplegic cerebral palsy, the corticospinal tracts of both sides have been affected. In these children, rhythmic modulation of the H-reflex during walking, which is suggested to be spinally regulated, is intact but the tonic depression of the H-reflex, which is assumed to be mediated through supraspinal centers is compromised. Therefore, it seems that the centrally driven modulation of the H-reflex is affected in these children [45]. This is one example in which understanding the underlying mechanisms is relevant for the development of behavioral specific interventions in individuals with motor dysfunction.

Comparison of the findings regarding the activity-dependent role of NMDA receptors at the level of the spinal cord and the importance of cortical input to the spinal cord strongly suggests that both peripheral and descending inputs are required for activity-dependent plasticity in the spinal cord. It is shown that these activity dependent eliminations of synapses are at least partly mediated by NMDA receptor activation. Recent investigations [46] have shown that the postsynaptic GluN2B subtypes of NMDA receptors play an important role for this elimination. The GluN2B-containing NMDA receptors are better conductors of Ca^2+^ into the cells. While it is understood that NMDA receptors mediate many activity-dependent changes during the early stages of spinal cord development, the exact mechanism by which NMDA receptors function to regulate development is unknown.

## 3. Activity-Dependent Plasticity in the Adult Spinal Cord

Unlike the literature on the developing spinal cord, much of our understanding of activity-dependent plasticity of the mature spinal cord comes from human studies. The H-reflex is a well-recognized and accepted method for investigating the function of the spinal circuits during various movements. For eliciting an H-reflex, an electrical stimulus (usually a single square-wave pulse with 1 ms duration) is applied to a peripheral nerve [47]. The largest sensory fibers (the Ia fibers), due to their axonal diameters are the first to be stimulated. These sensory afferents transmit the signal to the spinal cord and synapse both directly and indirectly onto alpha motoneurons. The resulting activation of the alpha motoneurons can be detected as a synchronized, coherent biphasic signal in the EMG activity of the corresponding muscle. For this reason, the H-reflex is regarded as an electrical analogue to the stretch reflex [48] (although there is considerable debate about this comparison). This reflex arc is nonetheless under the influence of descending drive and input from the periphery as well as other muscle spindles [15].

Applying this technique to any accessible mixed nerve will elicit the H-reflex in the corresponding muscles; however, this technique has been most widely used for examination of the soleus muscle due to the superficial location of its neural innervation. More importantly, the soleus is a crucial muscle for the control of posture and gait. Therefore, measuring the H-reflex in the soleus muscle is an appropriate model for studying the role that spinal circuits play in the control and modulation of a variety of bipedal movements. We would like to point out that the findings from this type of artificially induced reflex might be different than those of stretch reflexes. There are studies that show that modulations observed in the H-reflex are not present in the stretch reflex [49]. It is assumed that the H- and the stretch reflex are not equally sensitive to inhibitory mechanisms such as presynaptic inhibition. This difference can be partly explained by the fact that the H-reflex is temporally more synchronized than the stretch reflex and therefore, the temporal dispersion associated with the stretch reflex might render the Ia fibers less sensitive to presynaptic inhibition [50]. This idea is supported by the fact that repetitive discharge of Ia fibers reduces their susceptibility to presynaptic inhibition [50].

### 3.1. Short-Term and Long-Term Changes in the Synaptic Strength of the Reflex Arc

In adult humans, monkeys, and rats learning new skills is accompanied by temporary or permanent changes in the spinal cord, and these changes have been extensively studied with the stretch reflex or the H-reflex.

It is well accepted that there exists short-term task-dependent modulation of spinal reflexes, and this modulation does not immediately impose any structural or long-lasting functional change in spinal circuits. The prevailing notion is that synapse strength is altered in a task-specific manner. However, practicing the same task or stimulating the same pathway for an extended period of time (e.g., days or years) can result in long-term structural changes in spinal circuits. One example from the athletic area is the reflex regulation in dancers in whom the amplitude of the H-reflex is substantially lower than the normal population [51–53]. Presumably, these long-term changes may in fact weight the contribution of the corticospinal tract in modulating segmental inputs during highly skilled movement, with less weight given to the peripheral input of the muscle spindles.

To examine the induction of such long-term change in the adult spinal cord, an operant conditioning model of spinal reflexes has routinely been used. In this model of learning, a spinal reflex (stretch or H-reflex) is elicited, and the resulting EMG response is recorded. The amplitude of the reflex is presented to the subject as a feedback. A reward is provided if the reflex response is modulated in one particular direction (increase or decrease) as determined by the examiner. This reward encourages the animal (or human) to purposefully direct its behavior toward the desired reflex response. Operant conditioning has been extensively used for documenting changes in the input-output relationship of both the spinal stretch and the H-reflex. This model has provided a powerful tool for the investigation of spinal circuits as well as any morphological alterations in the motoneurons associated with learning. It is now well established that both animals and humans can similarly increase or decrease the amplitude of the stretch or H-reflex [54]. Typically, the plasticity in these circuits has consistently been shown to be nearly 150% increases in amplitude for those rewarded for increases, and nearly 50% decreases in reflex amplitude for those rewarded for decreases [55, 56].

### 3.2. Presynaptic Inhibition as One Method for Altering Synaptic Transmission

How do these changes in the reflex pathway occur and how do they become permanent?

For the efficacy of the synaptic transmission to change (either transiently or permanently), there are some mechanisms which act on the presynaptic terminals and some mechanisms which affect the postsynaptic terminal. Collectively, such presynaptic or postsynaptic alterations can increase or decrease the amplitude of EPSPs or inhibitory postsynaptic potentials (IPSPs). There is a variety of these mechanisms throughout the central nervous system which are involved in almost all activities of the CNS from learning and memory [57], habituation [58], and gating of pain signals [59] to the control of movement [60–63]. At the level of spinal motoneurons, both types of mechanisms exist and have role in the modulation of the H-reflex and stretch reflex in different movements [64–67]. In general, postsynaptic mechanisms that exert inhibition on alpha motoneurons result in these motoneurons being less responsive to any type of excitatory input. Presynaptic inhibitory mechanisms, on the other hand, can affect the input to the motoneurons without affecting the motoneurons intrinsic properties. This type of inhibition selectively inhibits one input to the motoneurons without affecting other inputs. Likewise, inhibition of the Ia-motoneuron synapses presynaptically can render the reflex gain lower (can reduce the amplitude of the reflex) without affecting the excitability status of the motoneurons. In this case, the normal activity of the muscle will be secured, while its reflexive contraction (and thus its selective control of incoming sensory information) can be independently reduced.

**Figure 1:**
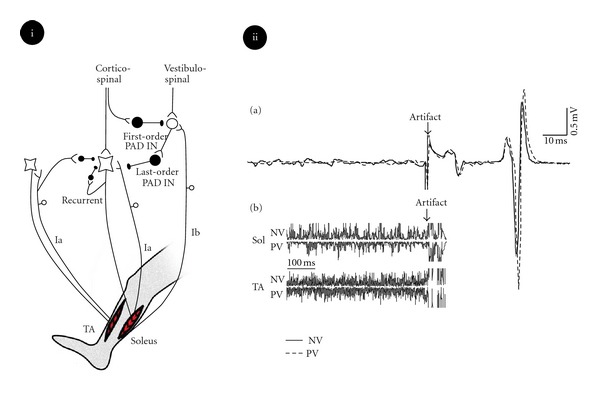
Presynaptic inhibition to Ia fibers. (i) Schematic diagram of different inputs to Ia afferents and alpha motoneurons. Proprioceptive input from Ia fiber can be selectively suppressed by presynaptic inhibition through PAD interneurons. The interneuron which makes axoaxonic connection with Ia fiber is GABAergic and regarded as last-order PAD IN. This interneuron is under the influence of an excitatory interneuron which is referred to as first-order PAD IN. This first-order PAD IN receives inputs from both descending tracts and from peripheral afferents [15]. In such a case, different inputs can interact to control the Ia input to motoneurons without affecting the intrinsic properties of motoneurons. (ii) During presynaptic inhibition, the normal activity of the muscle can remain unchanged, while the reflex gain reduces. In this example, standing with prism goggles (PV condition) suppressed the H-reflex in comparison to normal vision (NV) standing condition (a), while there was no change in the soleus and tibialis anterior muscle EMG activity (b). This is most likely due to the presynaptic inhibition of Ia fibers which spares the background activity of motoneurons. Part II adapted with permission from [16].

Frank and Fuortes (1957) were among the first to report that sensory inflow can indeed be suppressed without affecting the resting potential of the postsynaptic alpha motoneuron [68]. However, they did not provide a reasonable explanation on how the monosynaptic transmission can be manipulated without any change in the input level or any change in the resting potential of the postsynaptic cell. Later, Frank [69] suggested that there could be what he termed a “remote inhibition” meaning that the site of this inhibition is remote from the soma [70, 71]. The existence of this phenomenon was confirmed in subsequent research [72], but it was not well understood until the pioneering work of Eccles, who suggested that Ia afferent synaptic strength can be affected through axoaxonic GABAergic inhibitory connections [72, 73]. The prevailing hypothesis for the mechanism of presynaptic inhibition of Ia afferents is that the GABAergic receptors in the active zone of the primary afferent terminal (presynaptic Ia terminals) are being activated by interneurons of other sources (refer to Figure 1). Because both sides of this synaptic terminal are axons (Ia afferent and the interneurons), this specific type of synaptic connection was termed axoaxonic to address this phenomenon. These interneurons, while being activated, act GABAergically on the Ia terminals [74, 75]. Upon the opening of the GABA_A_ receptors in the Ia terminals, chloride ions leave the presynaptic terminal and thereby cause the active zone to depolarize. It is suggested that this GABAergic mechanism shunts the EPSP through GABA_A_ receptor activation, or directly affects the Ca^2+^ channels through GABA_B_ receptors [70, 76]. Without an influx of Ca^2+^, vesicle mobilization is impaired, decreasing the probability of neurotransmitter release from the afferent terminals [70, 77]. It was shown in the cat that the interneurons which mediate this primary afferent depolarization (PAD), are under the influence from both peripheral sources such as Ib volleys and Ia input from antagonistic muscles [73, 78, 79] and cutaneous afferents [80], as well as from the descending tracts such as rubrospinal tract [81] and corticospinal tract [82, 83]. How the nervous system affects these different pathways to reach the desired level of activity in literally thousands of motoneurons remains a mystery in neuroscience research.

### 3.3. Functional Significance of Presynaptic Inhibition

Presynaptic inhibition of Ia afferents is highly modifiable in response to postural changes [10] and motor tasks [84, 85]. Presynaptic modulation of Ia inflow could be a physiologic mechanism for adjusting the amount of feedback to the central nervous system.

Homonymous [86] as well as heteronymous [87] muscle afferents can presynaptically affect the sensory inflow of a given Ia afferent. These sources, due to their origin, are regarded as peripheral sources for presynaptic modulation. There are, on the other hand, centers in the brain (such as the red nucleus and vestibular nuclei) which can also affect the presynaptic terminals through their descending drive. Such a central influence on presynaptic interneurons can be collectively regarded as a central source for presynaptic modulation.

There is evidence to show that peripheral and central drives merge to the same common PAD interneurons [88] and therefore, these two sources can interact and integrate at the level of spinal cord [89]. Such an interaction can modify a reflexive activity that would elicit a large amplitude perturbation.

Taken together, it can be argued that adjusting the amount of presynaptic inhibition through the interaction of central and peripheral inputs has an important role in the execution of voluntary movements. For these reasons, it is now difficult to differentiate between reflexive and voluntary movements [90]. Whereas data indicate that presynaptic inhibition can significantly influence movement, it is not the only inhibitory mechanism in the spinal cord that has an effect on motor behavior. Other mechanisms such as postactivation depression [61], recurrent inhibition [91], and reciprocal inhibition [92, 93] all have functional roles in the control and execution of movement. However, prevailing evidence [16, 63, 80, 94–99] suggests that presynaptic inhibition has a critical role in the regulation of movement.

On the other hand, presynaptic inhibition has been repetitively shown to be modifiable in response to motor practice and learning new skills. In the following sections, we briefly review some key studies which have demonstrated short-term and long-term adaptations of the spinal circuits.

### 3.4. Goal Directed Changes in Presynaptic Inhibition of Ia Fiber Inputs to the Spinal Cord

During normal movement execution, such as changes in posture [100], movement initiation [101], and gait [102], presynaptic inhibition has been shown to be modulated. Besides the naturally occurring task specific modulation of presynaptic inhibition, the amount of presynaptic modulation expressed on spinal circuits is trainable. There is ample evidence in the literature to show that the amount of presynaptic inhibition can be purposefully changed. The experimental methods used to document this inhibition generally fall into operant conditioning of the reflexes and task-related feedback conditioning of the reflexes. It should be emphasized that none of these protocols exclusively target the PI circuits; rather, they exert various changes on spinal and/or even supraspinal circuits including alterations in presynaptic inhibition. However, both protocols can produce short-term as well as long-term changes in the neural circuits of the reflex pathway.

In operant-conditioning protocols, there seems to be a complex interaction of mechanisms involved in the induced plasticity including presynaptic modulation of the Ia terminals, specifically for the short-term adaptation phase [103]. While operant conditioning does not usually involve any specific task, there are protocols specially designed to modulate the H-reflex to fulfill some experimentally defined functional task. These task-related-feedback conditioning protocols usually provide feedback to the subject after each trial. Trimble and Koceja were the first to successfully implement such a functional protocol for short-term changes in spinal reflexes [97]. Their protocol involved a balance-control task in which subjects stood on a tilt board and were instructed to maintain their balance in a highly precarious posture. Applying an electrical stimulation for eliciting the H-reflex to the bilateral soleus muscles during this task produced enough ankle torque to destabilize the subjects during the trial. Over a single session of practice, subjects were able to learn to depress the H-reflex to minimize the destabilizing torque, as a strategy to maintain balance subsequent testing of the same subjects on a solid surface (normal upright standing) revealed that the H-reflex amplitude remained depressed for more than 30 minutes after the termination of the training session [97]. An ensuing study examined the effect of multisession training on the maintenance of the suppressed H-reflex. Two hours of H-reflex suppression training for three days significantly reduced the amplitude of the H-reflex which showed a trend to remain depressed for a longer period of time posttraining [98].

Such types of training-induced plasticity have also been observed in more complex movements. In a novel locomotion study, subjects were trained to walk backward on a treadmill for several weeks. In untrained subjects a large amplitude H-reflex was observed during the midswing phase of walking. Training progressively reduced the amplitude of the reflex. However, these changes in the reflex amplitude were not related to leg muscle motor evoked potentials (MEPs). It was suggested that the plasticity induced in the H-reflex circuits was heavily dependent on the presynaptic control of the inflow of sensory information [99]. It is interesting that a comparison of the results of studies using task-related-feedback conditioning with those using operant conditioning suggests that the two methods produce relatively the same percentage of change in the H-reflex. What remains to be determined, and may be an important distinction, is whether these two types of feedback result in the same types of functional and/or behavioral consequences. Studies using operant conditioning as a method for functional motor improvement have already been initiated, and thus far have provided promising results [104–106].

### 3.5. Practice Makes Permanence

In humans, it has been clearly established that long-term, repetitive activity produces changes in the reflex arc. For example, strength training has been shown to increase H-reflex amplitude; 14 weeks of muscle-specific heavy resistance training can increase the soleus H-reflex amplitude by 20% [107]. Research has also shown that ipsilateral resistance training increases the strength of both limbs, most likely due to neural adaptation, but that the H-reflex amplitude increases only in the trained side [108]. This finding supports the idea that direct increase in sensory inflow is necessary for the induction of plasticity in spinal circuits.

As another example, several studies have shown that the amplitude of the H-reflex is significantly reduced in trained dancers [51–53]. The reduction in the H-reflex amplitude is presumed to be caused by long-term performance of dance specific movements. Cocontraction of the lower limb muscles, which is frequently utilized in ballet dance, induces an increase in presynaptic inhibition, and causes a reduction in reciprocal inhibition. This activity-induced change in the H-reflex is most likely a part of the process of acquiring high-level skill and maintaining balance for dance-specific techniques. This reduction in response to peripheral sensory input could also be interpreted as an increase in a cortical role for the control of movement, and hence a more precise movement.

Taken together, these studies provide evidence that neural circuits can undergo long-lasting activity-induced plastic changes. However, these studies cannot unambiguously conclude that the plastic changes were induced solely in the spinal cord circuits. One possibility is that functional changes in these circuits are due to changes in descending drive rather than the spinal cord.

To determine whether the long-term changes occur at the spinal or supraspinal levels, Wolpaw and his colleagues examined the effect of operant conditioning on the stretch or the H-reflex in monkeys and human subjects [56, 109, 110]. Wolpaw and O'Keefe demonstrated both in monkeys and humans that the stretch reflex, as well as the H-reflex, can be down- or upregulated using operant conditioning. Wolpaw and colleagues also demonstrated that plasticity occurs in two distinct phases: an immediate (acute) phase which was observed in the same day of training (approximately 8–10% change) and a long lasting (approximately 1-2%/day for many days) phase. The acute phase was readily observed in the stretch reflex but not the long-loop reflexes which are assumed to involve higher centers such as the cortices. This immediate phase was temporary and diminished within a few hours after the termination of the training session. However, by continuing the training sessions for 4–6 months in humans and monkeys, respectively, the plasticity became more permanent and the modulation persisted for months after termination of the training sessions. Severing the spinal cord after the reflexes were up- or downregulated (in two different groups of monkeys) did not diminish the up- or downregulated reflex [110], supporting the idea that the plasticity had resided within the spinal circuits.

### 3.6. Central versus Peripheral Contribution for the Induction of Plasticity and Memory Formation in the Spinal Cord

Acute changes in spinal pathways are believed to be triggered by descending inputs. However, changes in the descending input over a long period of time can produce permanent changes in the spinal cord which are regarded as spinal fixation. Animals with partial transection of the spinal cord with an intact corticospinal tract are still able to volitionally up- or downregulate the H-reflex in an operant conditioning protocol [111]. However, spinal circuits can undergo plastic changes in response to exercise and skill acquisition which is not dependent on corticospinal drive. Operant conditioning is a specific type of memory formation and due to its nature (volitional alteration of the reflexes based on the feedback and reward) the descending input is an indispensable part of it. While the results of the studies on operant conditioning have provided valuable information and insight about memory formation in spinal circuits, conclusions from these studies should be interpreted with caution. First, it should be considered that during an operant-conditioning task, changes in the amplitude of the reflexes are not necessarily the *consequences* of a motor demand. Second, no functional tasks are involved during classical operant conditioning, which means that this type of conditioning may not be behaviorally relevant and these results do not translate to real-life situations.

Does the spinal cord have the ability to acquire new motor skills without the need of the descending drive for this skill acquisition?

Spinalized cats are indeed able to develop functional tasks despite the permanent loss of descending input [112, 113]. Such task-dependent modulation in segmental reflexes have also been observed in spinalized human patients as well [114]. Unfortunately there are few studies performed on normal human subjects to parsimoniously demonstrate changes in the spinal circuits, independent from descending drive. One obvious reason for this scarcity of information is the difficulty in differentiating the role of descending and peripheral inputs to the spinal cord. It is possible that a given *pattern* of sensory input (such as that generated by a specific task) may induce plastic changes in spinal circuits without the involvement of descending drive. In an excellent investigation, Meunier and colleagues [115] examined this possibility by training the subjects to perform two different types of cycling movements. In one group subjects performed a cycling exercise in which the resistance of the pedaling changed every 15 seconds, and they were asked to keep the cycling speed constant (e.g., complex task). In a second group, subjects performed the same task under constant pedaling resistance (e.g., simple task). It was shown that homosynaptic depression (the depression in the Ia transmission of sensory information after an immediate preceding stimulation) substantially changed only in the complex task group. Since homosynaptic depression is confined exclusively to the previously activated Ia fibers and there is no anatomical connection from the upper centers, investigators concluded that it was the *pattern of sensory inflow* that produced the change in synaptic efficacy between the Ia afferents and the alpha motoneurons. Again, understanding this discrepancy is extremely important for the improvement of modern rehabilitation techniques for spinal cord injury patients.

## 4. From Behavior to Cellular Events and Back

The exact mechanism of long-term activity-dependent changes in spinal circuits is not yet well understood. In operant-conditioning experiments designed to modulate the reflexes, initially the induced changes (in terms of the modulation of the reflexes) are reversible and will be abolished if training is discontinued. However, by continuing the task, the changes in the amplitude of the reflexes become permanent. In contrast, in mature animals, the transection of the corticospinal tract before or during the learning phase prevents the induction of long-term plastic changes [111, 116, 117]. Histological analysis of alpha motoneurons that have undergone permanent changes have shown morphological changes in the C and F terminals of the neurons [109] as well as changes in the size of the motoneurons, their input resistance, and axonal conduction velocity [118]. Future research should focus on the mechanisms that trigger these changes in motoneurons.

Learning a new skill is accompanied by a novel combination of muscle activity patterns that are temporally and spatially timed. These novel biomechanical configurations produce new sensory information feedback to the nervous system. Timely coupling of the EPSPs with action potentials has been shown to alter synaptic efficacy [119]. The backfiring of action potentials from the axon to the dendrites, if coincident with the EPSP, can affect the EPSP magnitude and potentially alter synaptic strength. A similar mechanism could exist in the spinal cord which affects the synaptic efficacy through the timed arrival of the sensory input coincident with descending commands. Interestingly, blocking NMDA receptors prevents the modulatory effect of action potential on the EPSPs. The studies on long-term potentiation (LTP) and long-term depression (LTD) could be used as evidence to show that such timely coupled inputs could lead to the consolidation of new skills in the spinal circuits. LTP and LTD have been experimentally induced in superficial dorsal horn [120], intermediate gray area [121], and ventral horn [122] of the spinal cord, and blocking NMDA receptors prevents LTP induction in these regions. Interestingly, EPSPs are not affected by NMDAR blockade, but blocking non-NMDA receptors substantially diminishes EPSPs. Therefore, under normal conditions, non-NMDA receptors appear to be predominantly responsible for the generation of EPSP's. Furthermore, it seems that NMDA receptors do not have a critical role in the maintenance of LTP, since blocking NMDA receptors after LTP induction has no effect on LTP. Conversely, blocking non-NMDA receptors after LTP induction substantially decreases LTP expression, demonstrating that non-NMDA receptors are necessary for the maintenance of LTP. Consistent with *in vitro* and *in vivo* animal studies, blocking NMDA receptors in human subjects using Dextromethorphan interferes with the acquisition of motor memory but does not impair motor memory recall [123].

The studies that have shown LTP in other areas of spinal cord have not investigated the mechanisms of LTP induction and maintenance, but it is unlikely that the role of NMDA receptors in LTP induction and maintenance is topographically distinct across spinal cord regions. A direct study between LTP and reflex regulation has not yet been reported however, it would be of value to examine the effect of NMDA receptor antagonists on the induction of H-reflex upregulation. If a behaving animal, treated with NMDA antagonist to the substantia gelatinosa, cannot upregulate the H-reflex, this might suggest the importance of the substantia gelatinosa on the memory capacity of the spinal cord for movement regulation. Such investigations on NMDA receptors might provide new advances for the restoration of spinal ability and motor control. Increasing the basic knowledge of activity-dependent plasticity throughout the life span of humans can substantially influence the treatment and rehabilitation methods used for various neurological conditions.

## 5. Concluding Remarks

Spinal circuits possess the ability for plastic changes to fulfill short- and long-term motor demands. These reversible changes in spinal circuits are typically accompanied by alterations in synaptic strength for acute adaptations, and by morphological and electrophysiological changes for long-term adaptations. Examining the modulation of spinal reflexes during different tasks has provided much of our understanding about activity-dependent plasticity in the spinal cord. During normal walking, for example, stretch reflexes are modulated differently compared with upright standing. The amplitude of the stretch reflex is not constant throughout the cycle of gait, and the phases of gait also affect the strength of the stretch reflex. Practicing a particular skill for extended periods of time can also affect the amplitude of the reflexes. Such changes in reflex gain have been shown to be associated with the degree of performance. For example, the ability of subjects to maintain a constant pedaling speed against varying the resistance during the bout of exercise was shown to be strongly correlated to the degree of H-reflex modulation [124]. These findings open the doors for seeking rehabilitation methods to specifically train reflexes with the aim of improving the function. During motor pathologies, such as spinal cord injury or brain damage, spinal reflexes still pose the ability to be modified [125]. It is only through goal-directed, precise rehabilitation strategies that potential plastic abilities of the spinal circuits can be used to regain function. Therefore, understanding the mechanisms and sites of plasticity within spinal circuits is essential for the development of new methods that can be used to regain spinal cord function, including the control of movement, after injury.  Table 1 summarizes the topics reviewed in this paper and provides a brief comparison about the factors which are involved in the plasticity of the developing and mature spinal cord.

## Figures and Tables

**Table 1:** Different aspects of activity-dependent spinal plasticity in the developing and a mature spinal cord, discussed in this paper.

	Developing	Mature
Cat-301	Increase in response to movement and sensory input to SC during critical period. Large nerve crush inhibits the expression of the antibody.	Not substantial. After the critical period, nerve crush does not affect the expression of the antibody.
NMDA receptors	Have role in the induction of synaptic plasticity. Probably have role in the induction of morphological changes. Have role in dendritic growth and retraction.	Blocking the receptors does not affect motoneuron morphology. These receptors are being eliminated from almost all parts of the spinal cord except for substantia gelatinosa. Likely do not have substantial role in reflex transmission.
Elimination	Substantial elimination during maturation. Cortical connections to the ipsilateral side of the spinal cord will be eliminated during maturation. Dendrites grow and retract. This is a model of non-Hebbian activity dependent process. At the neuromuscular junction, many synaptic connections are lost which results in muscle fibers from polyneural innervation to mononeural innervation [23, 24].	Synaptic connections mostly follow Hebbian process. Activity-dependent plasticity does not seem to eliminate synapses.
Sensory input	Sensory input is essential for developing spinal cord. Sensory information generated my movement seems to have role in the development of spinal synapses and circuits.	Have role in both transitional as well as permanent changes in the spinal circuits. Pattern of sensory input has been shown to have role in the induction of plastic changes.
Presynaptic modulation	Likely presynaptic inhibition exists in infants and is being modulated in response to movement. However, the role of presynaptic inhibition in the acquisition of new skills in newborn infants and children has not been extensively studied. Recent studies on mouse models have shown that undernourishment substantially decreases the amount of presynaptic inhibition [25]	Has important role in the modulation of reflex gain during different movements, at the initiation of movement, and for postural control. Skill acquisition (such as dance) can permanently change the amount of presynaptic inhibition. Presynaptic inhibition can also be increased or decreased through operant conditioning (absence of any functional task) and task-related feedback conditioning (presence of a functional task)
Descending influence	Has important role in the expression of Cat-301 and in the elimination of synapses through development.	Has important role in the induction of plastic changes in spinal cord during skill acquisition, operant conditioning and movement control and modulation of presynaptic inhibition, and other spinal mechanisms.
